# The complete mitochondrial genome of the Columbia lance nematode, *Hoplolaimus columbus*, a major agricultural pathogen in North America

**DOI:** 10.1186/s13071-020-04187-y

**Published:** 2020-06-22

**Authors:** Xinyuan Ma, Paula Agudelo, Vincent P. Richards, J. Antonio Baeza

**Affiliations:** 1 Department of Plant and Environmental Sciences, Clemson University, Clemson, SC 29634 USA; 2 Department of Biological Sciences, Clemson University, 132 Long Hall, Clemson, SC 29634 USA; 3 Smithsonian Marine Station at Fort Pierce, 701 Seaway Drive, Fort Pierce, Florida 34949 USA; 4 Departamento de Biología Marina, Facultad de Ciencias del Mar, Universidad Católica del Norte, Larrondo 1281, Coquimbo, Chile

**Keywords:** *Hoplolaimus*, Lance nematode, Ecdysozoa, Mitochondrial genome, Phylogeny, *de novo* assembly

## Abstract

**Background:**

The plant-parasitic nematode *Hoplolaimus columbus* is a pathogen that uses a wide range of hosts and causes substantial yield loss in agricultural fields in North America. This study describes, for the first time, the complete mitochondrial genome of *H. columbus* from South Carolina, USA.

**Methods:**

The mitogenome of *H. columbus* was assembled from Illumina 300 bp pair-end reads. It was annotated and compared to other published mitogenomes of plant-parasitic nematodes in the superfamily Tylenchoidea. The phylogenetic relationships between *H. columbus* and other 6 genera of plant-parasitic nematodes were examined using protein-coding genes (PCGs).

**Results:**

The mitogenome *of H. columbus* is a circular AT-rich DNA molecule 25,228 bp in length. The annotation result comprises 12 PCGs, 2 ribosomal RNA genes, and 19 transfer RNA genes. No *atp8* gene was found in the mitogenome of *H. columbus* but long non-coding regions were observed in agreement to that reported for other plant-parasitic nematodes. The mitogenomic phylogeny of plant-parasitic nematodes in the superfamily Tylenchoidea agreed with previous molecular phylogenies. Mitochondrial gene synteny in *H. columbus* was unique but similar to that reported for other closely related species.

**Conclusions:**

The mitogenome of *H. columbus* is unique within the superfamily Tylenchoidea but exhibits similarities in both gene content and synteny to other closely related nematodes. Among others, this new resource will facilitate population genomic studies in lance nematodes from North America and beyond.
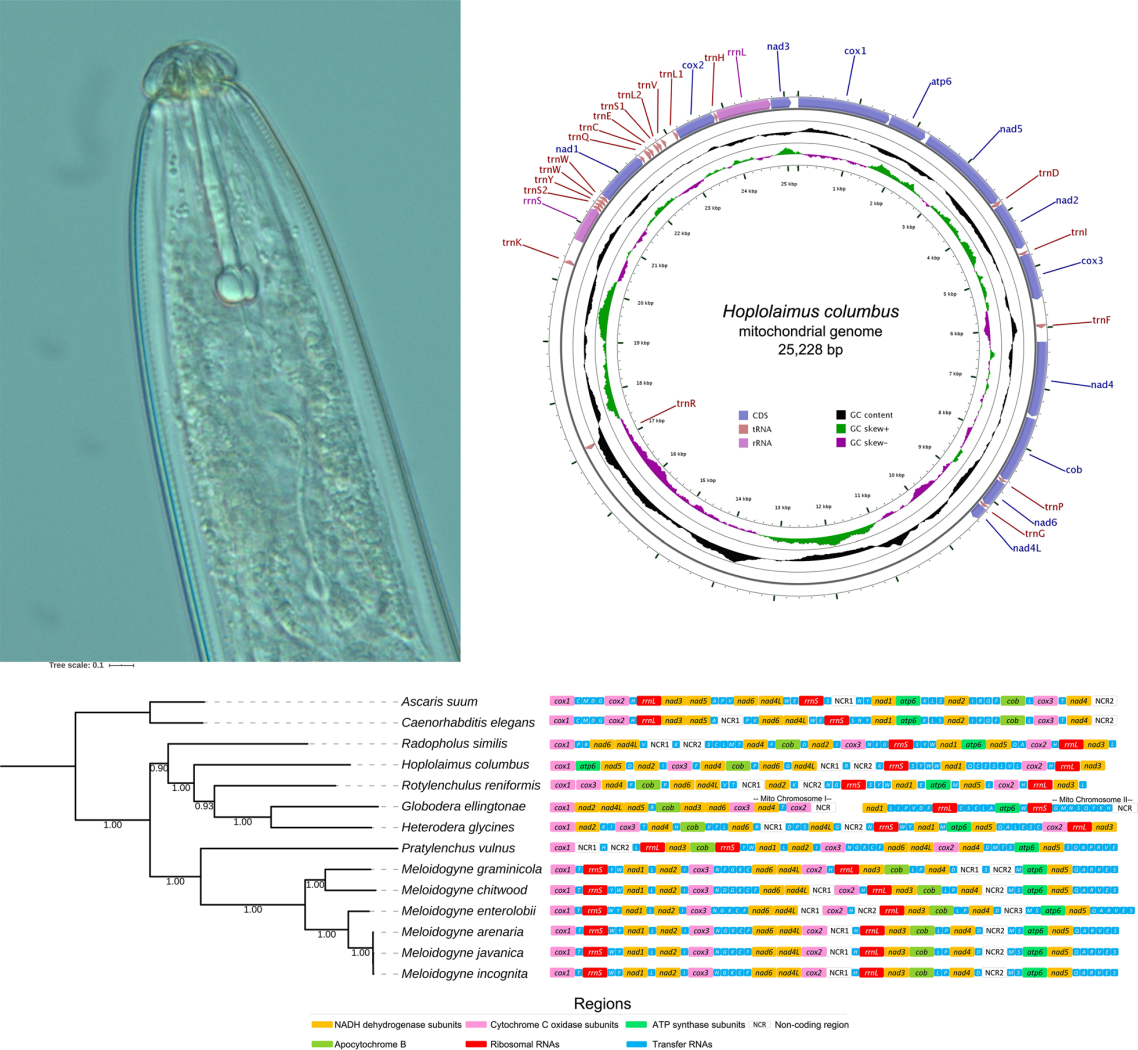

## Background

In the phylum Nematoda, plant-parasitic species can be distinguished from animal parasites as well as non-parasitic relatives because their mouthparts and stylet are well developed allowing them to penetrate sturdy plant cell walls while digging and feeding [1, 2]. A number of plant-parasitic nematodes are currently recognized as major pathogens of agricultural crops worldwide, which leads to more than 150 billion USD losses annually in the USA [3]. In a recent USA survey of agricultural pathogens, six main genera of plant-parasitic nematodes were recognized as serious crop threats [1]: cyst nematodes (*Heterodera* spp.); lance nematodes (*Hoplolaimus* spp.); root-knot nematodes (*Meloidogyne* spp.); lesion nematodes (*Pratylenchus* spp.); reniform nematodes (*Rotylenchulus* spp.); and dagger nematodes (*Xiphinema* spp.). Moreover, some of the above pathogens, like lance nematodes, can damage horticultural fields, golf courses, and turfgrasses. These plant-parasitic nematodes can also cause serious indirect environmental problems by favoring chemical overuse during nematode management [4].

Lance nematodes are all species of migratory ecto-endo parasites with a distinct cephalic region and a massive well-developed stylet [4]. According to the current taxonomical view that relies on a combination of molecular and morphological characters, lance nematodes belong to the class Chromadorea, infraorder Tylenchomorpha [2, 4–6]. They exhibit a wide range of hosts, including, among others, turf grasses, cereals, soybean, corn, cotton, sugar cane, and some trees [1, 4, 7]. They live in soil, feed on plant roots, move inside or around plant tissue, and destroy cortex cells, that can result in root necrotic lesions [8]. *Hoplolaimus columbus*, also known as the ‘Columbia lance nematode’, is considered among the most economically important species in the world [1]. This nematode was described as a new species from samples collected in Columbia, South Carolina, USA. Later, the same species was reported in the states of North Carolina, Georgia, Alabama, and Louisiana [8–10]. In the field, *H. columbus* is parasitic on cotton and soybean, on which pathogenicity has been demonstrated; production losses for cotton are typically 10–25%, and losses for soybean can be as high as 70% in the southeastern USA [11–14]. Although *H. columbus* has been found in some Asian countries [2], there are no reports yet of crop damage in the region. *Hoplolaimus columbus* belongs to the subgenus *Basirolaimus* together with 17 other nematode species [2]. Species in the subgenus *Basirolaimus* have been reported in Asian countries, including China, India and Japan [2]. Nonetheless, the only species in the subgenus *Basirolaimus* so far recognized as a major agricultural pathogen is *H. columbus* (i.e. in the USA) [2, 15, 16]. Considering its wide distribution and damage to crops, a better genomic understanding of *H. columbus* would prove helpful to understand its population genetic structure and effects, or the lack thereof, on commercially relevant crops.

Morphological characteristics alone have limited function to distinguish among closely related species in the genus *Hoplolaimus* given the remarkable similarity of internal and external organs and body parts among closely related species [5, 7]. Molecular markers have been shown to be useful for species identification and for understanding phylogenetic relationships and population genetics in different species of lance nematodes [5, 15–17]. Although previous work has provided valuable insights for nematode phylogeny [18–21], it has been noticed that short nuclear and/or mitochondrial gene markers are sometimes uninformative for revealing fine to moderate population genetic structure within a species [22]. This shortcoming can be addressed by developing genomic resources in this relevant group of lance nematodes. Although the genomes of a number of plant-parasitic nematodes have been sequenced and analyzed before [23–28], no genomic resources exist for lance nematodes, yet.

In this study, we *de novo* sequenced and assembled the complete mitochondrial genome of the Columbia lance nematode *H. columbus*. Other than annotating and providing a detailed description of the mitochondrial chromosome in this crop pathogen, we used protein-coding genes to explore phylogenetic relationships among plant-parasitic nematodes belonging to the class Chromadorea, superfamily Tylenchoidea.

## Methods

### Collection of specimens, DNA extraction and whole-genome amplification

Soil samples containing specimens of *H. columbus* were collected from the Edisto Research Center in Blackville, South Carolina (33°21’56.2”N, 81°19’46.9”W) and transported to Clemson University for further study. In the laboratory, nematodes were first extracted from soil samples using the sugar centrifugal flotation method [29]. A few fixed specimens were then identified using diagnostic key characters under an optical microscope [30]. Next, live nematodes (*n* = 9) were submerged into distilled water, starved for two weeks, and placed in a 3% hydrogen peroxide solution (Aaron Industry, Clinton, SC, USA) for 5 min before washing them in distilled water three times to eliminate potential microorganisms inhabiting their surface. Then, the same nematodes were placed separately in DNA Away solution (Molecular BioProducts Inc., San Diego, CA, USA) to eliminate potential DNA and DNase contamination and washed three times using PCR-grade water. Total DNA from each *H. columbus* specimen was extracted using a Sigma-Aldrich extract-N-Amp kit (XNAT2) (Sigma-Aldrich, St. Louis, MO, USA). The whole genome size of *H. columbus* was estimated to be ~300 million bp using flow cytometry [31, 32]. Whole-genome amplification (WGA) of each individual nematode was then performed using an Illustra Ready-To-Go GenomiPhi V3 DNA amplification kit (GE Healthcare, Chicago, IL, USA) following the manufacturer’s instructions. Three WGA replicates per nematode were performed, and the one with the highest DNA concentration tested using a Qubit fluorometer (Invitrogen, Carlsbad, CA, USA) was selected for the next generation sequencing library preparation.

### Library preparation and whole genome shotgun sequencing

The Nextera XT kit (Illumina, San Diego, CA, USA) was used for library preparation using the manufacturer’s instructions. Library concentration and fragment size distribution after library preparation were determined using a Qubit fluorometer (Invitrogen, Carlsbad, CA, USA) and a Bioanalyzer 2100 (Agilent Technologies, Santa Clara, CA, USA), respectively. Sequencing was conducted in an Illumina MiSeq with the v3 chemistry kit. A total of ~56 million reads (paired-end 300 bp) were generated and 98.11% of these reads were of high-quality. Approximately 13 Gb of sequence data had a quality score (Q-score) > 30.

### Mitochondrial genome assembly and annotation

Two assembly methods were employed to reconstruct the mitochondrial genome of *H. columbus*. The first method employed the program NOVOPlasty 2.7.2 [33]. Reads were trimmed using Trimmomatic-0.36 [34] and assembled in NOVOPlasty using the following options: K-mer = 31; insert range = 1.6; and insert range strict = 1.2. A partial *cox*1 gene sequence belonging to *H. columbus* available on GenBank (KP864628) was used as a ‘seed’ during the assembly. A total of 110,852 reads were used for the final assembly that generated a circular DNA molecule with an average coverage of 1,476x. The second method used the programs MIRA and MITObim (mitochondrial baiting and iterative mapping) [35, 36]. The parameter settings for mitochondrial genome assembly using this second strategy were: NW:mrnl = 0; AS:nop = 1; SOLEXA_SETTINGS; CO:msr = no. After 15 iterations, MITObim assembled the mitochondrial genome of *H. columbus*. Mitochondrial genome chromosomes assembled using the two methods above were identical to each other.

After mitochondrial genome assembly, protein-coding genes (PCGs) and non-coding regions were predicted using the invertebrate mitochondrial code (genetic code 5) on the MITOS web server (http://mitos.bioinf.uni-leipzig.de/index.py) [37]. Annotation curation and start + stop codon corrections were conducted using the ExPASy translate tool (https://web.expasy.org/translate/) [38]. Secondary structures of tRNA genes were predicted using MiTFi [39] as implemented in MITOS and depicted using the web server FORNA (http://rna.tbi.univie.ac.at/forna/) [40]. For the *rrnS* and *rrnL* genes, locations were first detected using MiTFi. Then, the entire sequence of each rRNA gene was predicted using NCBI BLAST comparisons with other nematode *rrnS* and *rrnL* sequences available in GenBank. Codon usage of the different PCGs was examined using Sequence Manipulation Suite (SMS) (http://www.bioinformatics.org/sms2/index.html) [41]. The entire mitochondrial genome was depicted using CGView Server (http://stothard.afns.ualberta.ca/cgview_server/) [42].

Two relatively long non-coding regions found in the mitochondrial genome of *H. columbus* were analyzed in detail. Microsatellite sequences in these regions were detected using Microsatellite Repeats Finder (http://insilico.ehu.es/mini_tools/microsatellites/) [43], tandem repeats were detected using Tandem Repeats Finder 2016 v4.09 (https://tandem.bu.edu/trf/ trf.basic.submit.html) [44], and putative hairpin structures were predicted and visualized in the RNAFold webserver (http://rna.tbi.univie.ac.at//cgi-bin/RNAWebSuite/RNAfold.cgi) [45].

### Mitophylogenomics in the superfamily Tylenchoidea

The phylogenetic analysis included full mitochondrial genomes belonging to a total of 14 nematode species in the class Chromadorea, of which 12 were plant-parasitic nematodes in the superfamily Tylenchoidea (Table 1). *Caenorhabditis elegans* (non-parasitic) and *Ascaris suum* (animal-parasitic) were used as outgroup terminals in our phylogenetic analysis. Each of a total of 12 PCGs (see results) was first aligned using MAFFT version 7 [46] and output files converted into Phylip format using the web server Phylogeny.fr [47, 48]. Then, poorly aligned positions in each of the 12 PCG sequence alignments were trimmed using BMGE (block mapping and gathering with entropy) [49]. SequenceMatrix [50] was used to concatenate all 12 PCG alignments in the following order: *atp6-cox*1*-cox*2*-cox*3*-cytb-nad*1*-nad*2*-nad*3*-nad*4*-nad*4L*-nad*5*-nad*6. The GTR + G nucleotide substitution model (Additional file 1: Table S1) selected using SMS (smart model selection) (http://www.atgc-montpellier.fr/sms/) [51] was used for maximum likelihood (ML) phylogenetic analysis conducted on the web server IQ-Tree (http://www.iqtree.org/) [52] with the default settings but enforcing the GTR + G model of nucleotide substitution. A total of 100 bootstrap replicates were employed to explore support for each node in the resulting phylogenetic tree that was depicted using the web server iTOL (Interactive Tree of Life) (https://itol.embl.de/) [53].

**Table 1:** Species used for phylogenetic analyses and protein-coding gene order survey in this study

Species	GenBank ID	Size (bp)
*Ascaris suum*	HQ704901.1	14,311
*Caenorhabditis elegans*	NC_001328.1	13,794
*Globodera ellingtonae* (I)	KU726971.1	17,757
*Globodera ellingtonae* (II)	KU726972.1	14,365
*Heterodera glycines*	HM640930.1	14,915^a^
*Hoplolaimus columbus*	MH657221	25,228
*Meloidogyne arenaria*	NC_026554.1	17,580
*Meloidogyne chitwoodi*	KJ476150.1	18,201
*Meloidogyne enterolobii*	KP202351.1	17,053
*Meloidogyne graminicola*	KJ139963.1	19,589
*Meloidogyne incognita*	KJ476151.1	17,662
*Meloidogyne javanica*	NC_026556.1	18,291
*Radopholus similis*	FN313571.1	16,791
*Rotylenchulus reniformis*	CM003310.1	24,572
*Pratylenchus vulnus*	NC_020434.1	21,656

^a^Incomplete genome

## Results and discussion

**Fig. 1:**
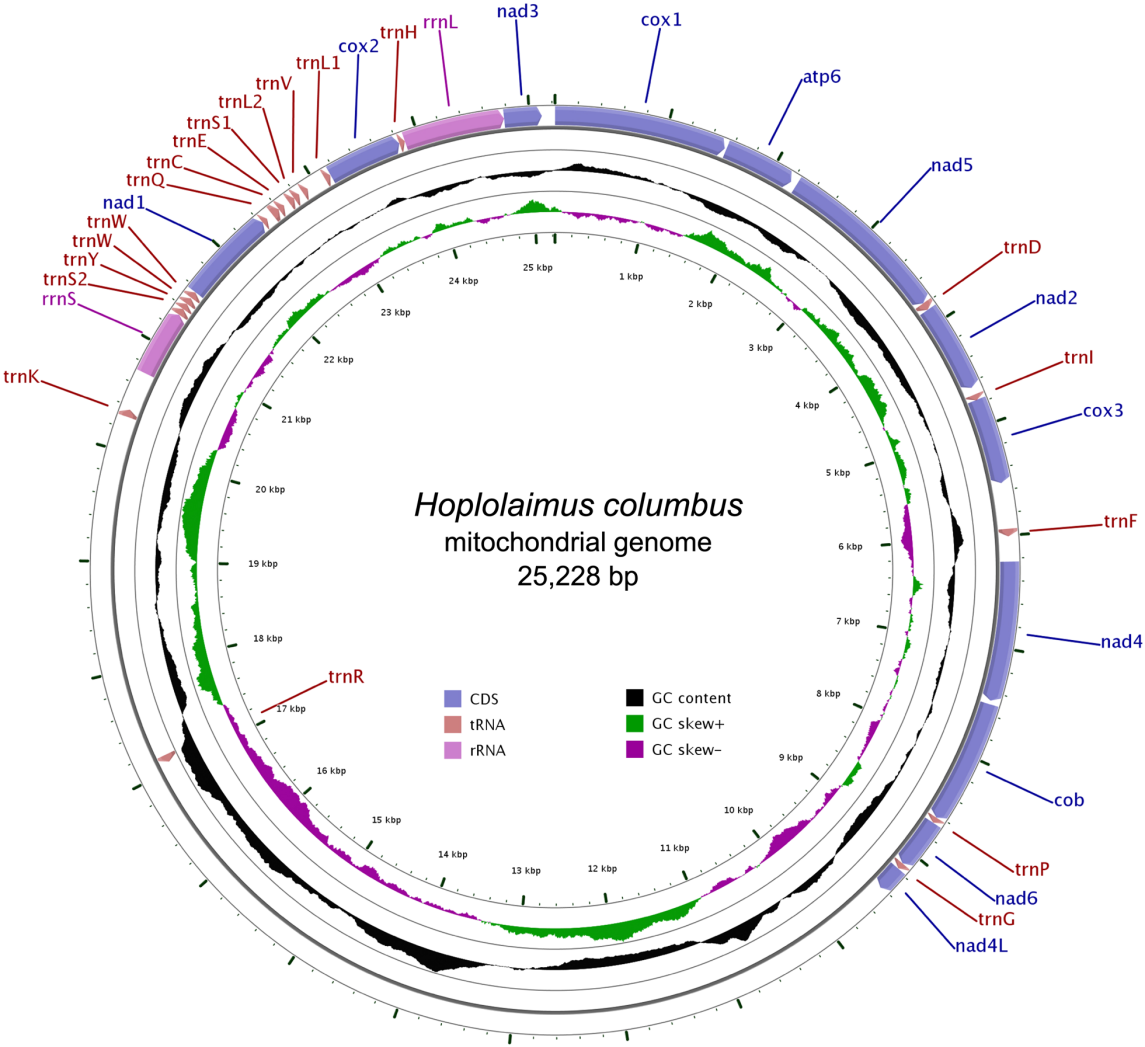
Circular genome map of *Hoplolaimus columbus* mitochondrial DNA. The map is annotated and depicts 12 protein-coding genes (PCGs), 2 ribosomal RNA genes (*rrnS* (*12S* ribosomal RNA) and *rrnL* (*16S* ribosomal RNA)) and 19 transfer RNA (tRNA) genes. The inner circle depicts GC content along the genome. The putative non-coding region likely involved in the initiation of the mitogenome replication is not annotated

The complete mitogenome of *H. columbus* was *de novo* assembled into a closed-circular DNA molecule of 25,228 bp in length (GenBank: MH657221; Fig. 1). The nucleotide composition of the entire mitochondrial genome was A = 28.46% (*n* = 7179 bp), T = 46.12% (*n* = 11,634 bp), C = 8.45% (*n* = 2132 bp), and G = 16.97% (*n* = 4281 bp). We also observed one R (position 3768, purine, A or G) and one Y (position 10529, pyrimidine, C or T). The mitogenome was strongly biased towards A + T (74.57%). The GC skew ((G - C)/(G + C)) and AT skew ((A - T)/(A + T)) values were 0.3351 and -0.2369, respectively. The mitogenome of *H. columbus* comprises 12 protein-coding genes (PCGs), 19 transfer RNA genes, 2 ribosomal RNA genes, and 2 large non-coding regions. The *atp8* gene was missing in the assembled mitogenome in agreement to that reported for other plant-parasitic nematodes [25–28] (Table 2).

**Table 2:** Gene annotation and arrangement in the mitochondrial genome of *Hoplolaimus columbus* from South Carolina, USA

Name	Type	Start	Stop	Length	Start codon	Stop codon	Direction	Anticodon	Intergenic space
*cox*1	Protein	1	1536	1536	TTA	TAG	Forward		116
*atp*6	Protein	1551	2192	642	ATT	TAG	Forward		14
*nad*5	Protein	2248	3788	1541	TTA	TT	Forward		55
*trnD*	tRNA	3789	3858	70			Forward	GUC	0
*nad*2	Protein	3859	4650	792	TTA	TAG	Forward		0
*trnI*	tRNA	4715	4770	56			Forward	GAU	64
*cox*3	Protein	4766	5533	768	TTA	TAA	Forward		− 5
*trnF*	tRNA	5941	6009	69			Forward	GAA	407
*nad*4	Protein	6232	7476	1245	TTG	TAA	Forward		222
*cob*	Protein	7509	8618	1110	ATA	TAG	Forward		32
*trnP*	tRNA	8619	8672	54			Forward	UGG	0
*nad*6	Protein	8673	9122	450	TTA	TAA	Forward		0
*trnG*	tRNA	9123	9179	57			Forward	UCC	0
*nad*4L	Protein	9182	9412	231	TTA	TAG	Forward		2
*NCR1*									
*trnR*	tRNA	17145	17074	72			Reverse	UCG	7661
*NCR2*									
*trnK*	tRNA	20303	20371	69			Forward	UUU	3157
*rrnS*	rRNA	20720	21317	598			Forward		348
*trnS2*	tRNA	21318	21380	63			Forward	UGA	0
*trnY*	tRNA	21380	21435	56			Forward	GUA	-1
*trnW*	tRNA	21436	21496	61			Forward	UCA	0
*trnW*	tRNA	21502	21561	60			Forward	CCA	5
*nad*1	Protein	21563	22422	860	TTA	GT	Forward		1
*trnQ*	tRNA	22423	22472	50			Forward	UUG	0
*trnC*	tRNA	22532	22604	73			Forward	GCA	59
*trnE*	tRNA	22606	22661	56			Forward	UUC	1
*trnS1*	tRNA	22714	22771	58			Forward	UCU	52
*trnL2*	tRNA	22772	22827	56			Forward	UAA	0
*trnV*	tRNA	22864	22917	54			Forward	UAC	36
*trnL1*	tRNA	23095	23150	56			Forward	UAG	177
*cox*2	Protein	23154	23823	670	TTA	T	Forward		3
*trnH*	tRNA	23824	23875	52			Forward	GUG	0
*rrnL*	rRNA	23876	24776	901			Forward		0
*nad*3	Protein	24777	25112	336	ATT	TAA	Forward		0

The PCGs in the mitochondrial genome of *H. columbus* contained 10,811 nucleotide residues: A = 2712 (26.64%); T = 5210 (51.17%); C = 693 (6.81%); and G = 1565 (15.37%). The strong A + T bias (77.81%) was within the known range reported for mitochondrial genomes in the superfamily Tylenchoidea [25–28]. Among the 12 PCGs, the 2 longest genes were *nad*5 (1541 bp) and *cox*1 (1536 bp), and the 2 shortest genes were *nad*4L (213 bp) and *nad*3 (336 bp) (Table 2). There were 8 genes that used the start codon TTA (*cox*1, *cox*2, *cox*3, *nad*1, *nad*2, *nad*4L, *nad*5 and *nad*6). Both *atp*6 and *nad*3 genes used the start codon ATT. The rather unusual start codons TTG of *nad*4 and ATA of *cob* have also been reported in other nematode species [25, 27]. Most of the PCGs used the complete stop codon TAG (*cox*1, *atp*6, *nad*2, *cob* and *nad*4L) or TAA (*cox*3, *nad*4, *nad*6 and *nad*3). The exceptions were three genes with incomplete stop codons; *nad*5(TT); *nad*1(GT); and *cox*2(T) (Table 2). The most frequently used codons in the PCGs were TTT (Phe, *n* = 601 time used, 17.72% of the total), TTA (Leu, *n* = 398, 11.74%), ATT (Ile, *n* = 262, 7.73%), ATA (Met, *n* = 139, 4.10%), AAT (Asn, *n* = 135, 3.98%), TAT (Tyr, *n* = 145, 4.28%). Less frequently used codons included GCG (Ala, *n* = 1, 0.03%), CGC (Arg, *n* = 1, 0.03%), ACG (Thr, *n* = 1, 0.03%), CAC (His, *n* = 2, 0.06%), and TCG (Ser, *n* = 2, 0.06%) (Table 3).

**Table 3:** Codon usage analysis of PCGs in the mitochondrial genome of *Hoplolaimus columbus* from South Carolina, USA

AmAcid	Codon	Number	/1000	Fraction	AmAcid	Codon	Number	/1000	Fraction
Ala	GCG	1	0.29	0.02	Pro	CCG	3	0.88	0.05
	GCA	11	3.24	0.17		CCA	13	3.83	0.23
	GCT	43	12.68	0.66		CCT	31	9.14	0.55
	GCC	10	2.95	0.15		CCC	9	2.65	0.16
Cys	TGT	20	5.9	0.77	Gln	CAG	16	4.72	0.47
	TGC	6	1.77	0.23		CAA	18	5.31	0.53
Asp	GAT	65	19.17	0.94	Arg	CGG	8	2.36	0.3
	GAC	4	1.18	0.06		CGA	7	2.06	0.26
Glu	GAG	37	10.91	0.43		CGT	11	3.24	0.41
	GAA	50	14.74	0.57		CGC	1	0.29	0.04
Phe	TTT	601	177.23	0.96	Ser	AGG	37	10.91	0.12
	TTC	23	6.78	0.04		AGA	82	24.18	0.26
Gly	GGG	38	11.21	0.19		AGT	68	20.05	0.22
	GGA	67	19.76	0.34		AGC	3	0.88	0.01
	GGT	85	25.07	0.42		TCG	2	0.59	0.01
	GGC	10	2.95	0.05		TCA	30	8.85	0.1
His	CAT	49	14.45	0.96		TCT	80	23.59	0.26
	CAC	2	0.59	0.04		TCC	10	2.95	0.03
Ile	ATT	262	77.26	0.94	Thr	ACG	1	0.29	0.02
	ATC	16	4.72	0.06		ACA	17	5.01	0.27
Lys	AAG	45	13.27	0.35		ACT	43	12.68	0.67
	AAA	82	24.18	0.65		ACC	3	0.88	0.05
Leu	TTG	101	29.78	0.17	Val	GTG	17	5.01	0.07
	TTA	398	117.37	0.68		GTA	76	22.41	0.33
	CTG	7	2.06	0.01		GTT	118	34.8	0.52
	CTA	29	8.55	0.05		GTC	18	5.31	0.08
	CTT	43	12.68	0.07	Trp	TGG	36	10.62	0.44
	CTC	5	1.47	0.01		TGA	45	13.27	0.56
Met	ATG	36	10.62	0.21	Tyr	TAT	145	42.76	0.93
	ATA	139	40.99	0.79		TAC	11	3.24	0.07
Asn	AAT	135	39.81	0.98	End	TAG	5	1.47	0.56
	AAC	3	0.88	0.02		TAA	4	1.18	0.44

**Fig. 2:**
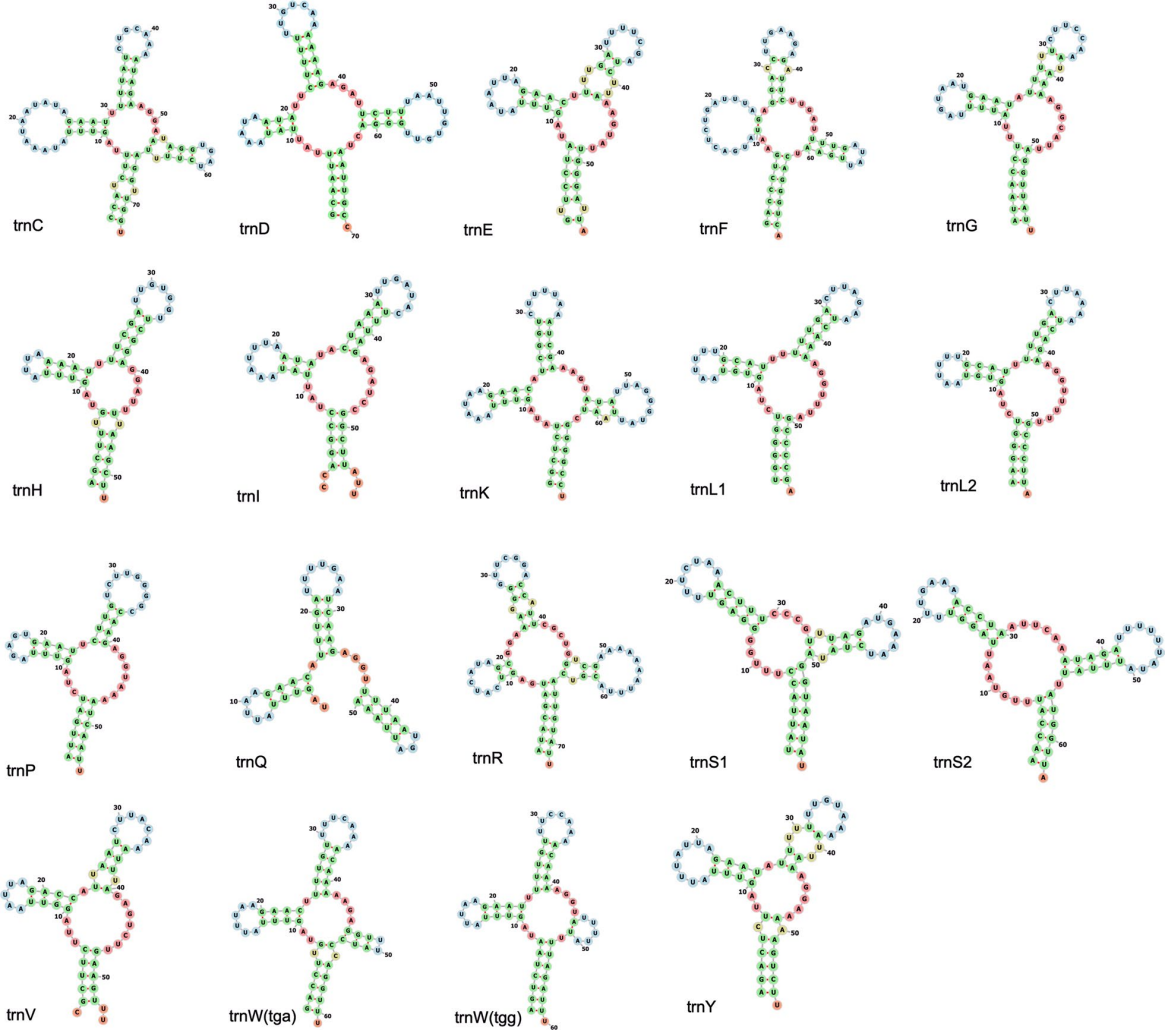
Secondary structure of tRNAs in the mitochondrial genome of *Hoplolaimus columbus* predicted by MITFI and FORNA

According to the prediction by MiTFi, the mitogenome of *H. columbus* comprises 19 tRNAs genes, ranging in length from 50 bp (*trnQ*) to 73 bp (*trnC*), including 2 *trnW* genes with different anticodons (UCA and CCA). Most of the tRNA genes encoded in the same direction as the PCGs and the two rRNA genes (*rrnS* and *rrnL*), except for the *trnR* gene which encoded in the opposite direction. There were 4 tRNA genes missing: *trnA*, *trnM*, *trnN* and *trnT.* Structure predictions of the different tRNAs are shown in Fig. 2. Most often, nematode tRNAs do not exhibit a regular canonical cloverleaf structure, either lacking the T-arm or missing both arms [54, 55]. In *H. columbus*, variable loops were found on the acceptor stem (*trnC* and *trnE*), on the T-stem (*trnC*, *trnR*, *trnS1* and *trnV*), and on the anticodon arm (*trnE*, *trnF*, *trnG*, *trnR*, *trnV* and *trnY*). The T-arm was missing in *trnE*, *trnG*, *trnH*, *trnL1*, *trnL2*, *trnP*, *trnV* and *trnY*. The D-arm was missing in *trnS1* and *trnS2*. The predicted structure of the *trnW(tga)* gene had a T-stem but no a T-loop.

The *rrnS* and *rrnL* genes identified in the mitochondrial genome of *H. columbus* were 598 bp and 901 bp nucleotide long, respectively (Fig. 1). The *rrnS* gene was located between *trnK* and *trnS2*. The *rrnL* gene was located next to *nad*3, between *rrnL* and *nad*3, in agreement to that reported for the mitogenomes of *Pratylenchus vulnus*, *Meloidogyne chitwoodi* and *M. incognita*. The overall nucleotide composition of the *rrnS* gene was A = 30.10%, T = 39.96%, C = 11.37%, and G = 21.57%, and that of the *rrnL* gene was A = 32.41%, T = 45.84%, C = 7.33%, and G = 14.43%.

Gene overlaps (6 bp in total) were found in 2 gene junctions: *trnI-cox*3 (5 bp) and *trnS2-trnY* (1 bp) (Table 2). In turn, relatively short intergenic spaces ranging from 1 to 116 bp were found in 12 gene junctions. Relatively long intergenic spaces were observed in the gene junctions *cox*3*-trnF* (407 bp), *trnF-nad*4 (222 bp), and *trnV-trnL1* (177 bp). Microsatellite repeats were detected in some of the above intergenic spaces (Additional file 1: Table S2). Cases of both overlaps and long intergenic spaces have been reported in mitogenomes of plant-parasitic nematodes [25–28].

Two long non-coding regions were identified, which might be useful in the future for nematode population genetics. One long non-coding region was located between the *nad*4L and *trnR* genes (NCR1, 7661 bp), and the second one was located between the *trnR* and *trnK* genes (NCR2, 3157 bp) (Fig. 1, Table 2). Long non-coding regions > 4000 bp have been reported in other plant-parasitic nematodes such as *Pratylenchus vulnus* (6847 bp), *Meloidogyne chitwoodi* (5404 bp), and *Meloidogyne incognita* (4097 bp), but *H. columbus* has the longest non-coding regions reported so far. The two regions were heavily A + T rich with an overall base composition of A = 29.79%, T = 39.86%, C = 10.90%, and G = 19.44% in NCR1, and A = 28.10%, T = 50.71%, C = 5.35%, and G = 15.84% in NCR2. Microsatellite repeats were detected in the two NCRs (Additional file 1: Table S3). Tandem repeat finder detected 13 repeats in NCR1 (the longest consensus size of a repeat was 237 bp, and the shortest one was 34 bp long) and 5 repeats in NCR2 (the longest consensus size of a repeat was 23 bp, and the shortest one was 18 bp) (Additional file 1: Table S4). No tandem repeat was found in other shorter intergenic spaces. Secondary structure prediction analysis using RNAFold detected a large number of hairpin structures in the two long NCRs (Additional file 2: Figure S1). Furthermore, a large number of microsatellite sequences were detected in the two non-coding regions (*n* = 104 and 72 in NCR1 and NCR2, respectively). Altogether, the observed high A + T rich nucleotide content, tandemly repeated sequences, and predicted hairpin secondary structures suggest that these two NCRs are possibly involved in the initiation of replication in the mitochondrial genome of *H. columbus*; all these features have been observed in the putative mitochondrial genome control region/D-loop of other invertebrates [56–60].

**Fig. 3:**
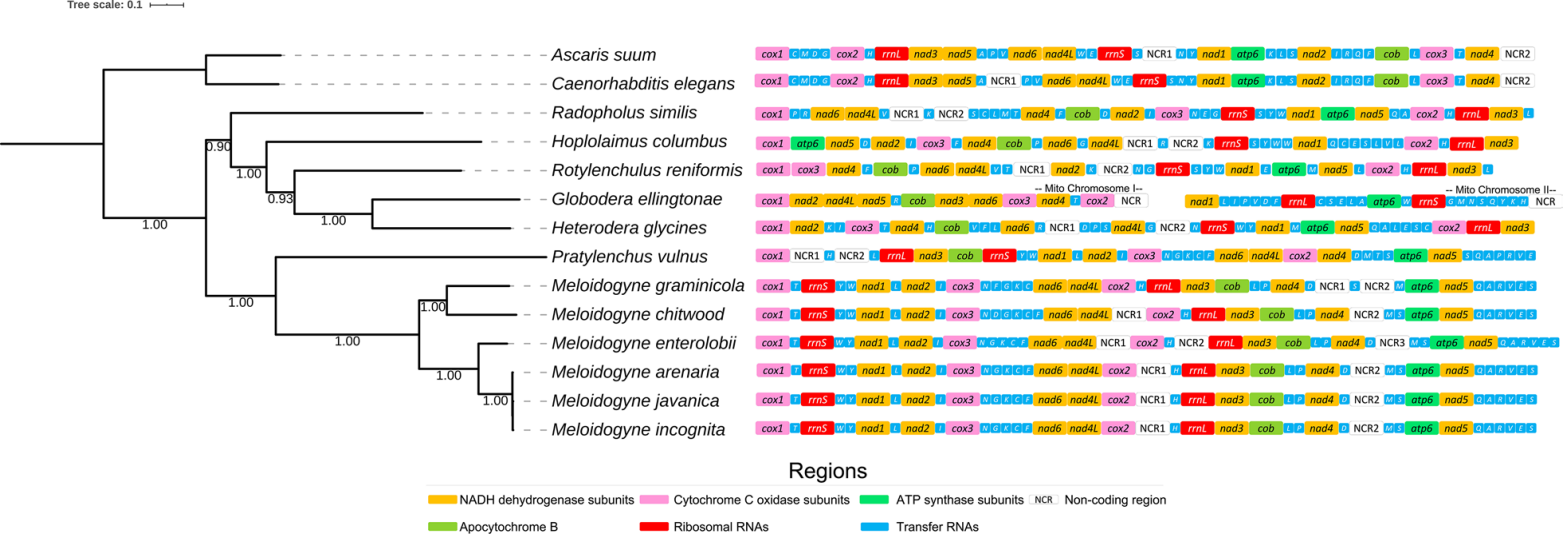
Mitochondrial gene synteny and phylogenetic analysis of *Hoplolaimus columbus* and related species. Phylogenetic tree obtained from Maximum Likelihood analysis was based on a concatenated alignment of nucleotides of the 12 protein-coding genes that presented in accessible mitochondrial genomes of plant-parasitic nematodes in the class Chromodorea, superfamily Tylenchoidea. In the analysis, *Caenorhabditis elegans* and *Ascaris suum* were used as the outgroup. Numbers at the branches represent bootstrap values. The optimal molecular evolution model estimated with SMS was the GTR model for all 12 partitions

The ML phylogenetic analysis (Fig. 3) confirmed the monophyly of the superfamily Tylenchoidea and placed *H. columbus* in a monophyletic clade together with *Radopholus similis*, *Rotylenchulus reniformis*, *Heterodera glycines* and *Globodera ellingtonae*, in agreement with previous molecular phylogenies [24–28]. Our results also supported the position of *H. columbus* as belonging to the family Hoplolaimidae. Moreover, the analysis revealed *Pratylenchus vulnus* to be sister to the genus *Meloidogyne*, and all species belonging to the genus *Meloidogyne* clustered together into a well-supported monophyletic clade. De Ley & Blaxter [6] recently suggested to classify Meloidogininae as a fully separate family based on the *SSU* rDNA phylogenies, and their view is supported by our mitophylogenomic analysis.

The synteny of protein-coding genes, ribosomal RNA genes, and non-coding regions observed in *H. columbus* was compared with that of other species in the same superfamily Tylenchoidea with completely annotated mitogenomes available in GenBank (Fig. 3). The mitogenome synteny of *Rotylenchulus reniformis* was not available in GenBank and was predicted in this study using the web server MITOS. A unique gene order was found in *H. columbus*, and this order is somewhat similar to that reported for other species in the same superfamily (Fig. 3). A visual comparison between phylogenetic relatedness and gene synteny also suggests that synteny might represent a useful phylogenetic character in this clade; a correlation between phylogenetic relatedness and gene synteny was observed in the studied plant-parasitic nematodes (Fig. 3) although variability is relatively high considering that the comparison was made among different genera belonging to the superfamily Tylenchoidea.

## Conclusions

This study *de novo* assembled, for the first time, the mitochondrial genome of *H. columbus*, a result that also represented the first mitochondrial genome description for the genus *Hoplolaimus*. The mitogenome of *H. columbus* had a relatively large size compared to that of other plant-parasitic nematodes, exhibits long non-coding regions, and has a unique gene order within the superfamily Tylenchoidea. The mitophylogenomic analysis also agreed with a previous phylogenic hypothesis established using the *SSU* rDNA marker, and confirmed the taxonomic relationships among species in the superfamily Tylenchoidea. Ultimately, we envision that this new genomic resource in *H. columbus* will help to improve our knowledge about the biology and population genetics of this economically and ecologically relevant agricultural pathogen both in Asia and North America

## Supplementary information

**Additional file 1: Table S1.** Model selection for phylogenetic analysis by smart model selection (SMS). **Table S2.** Microsatellite repeats in intergenic spaces. **Table S3.** Microsatellite repeats in non-coding regions. **Table S4.** Tandem repeats in non-coding regions.

**Additional file 2: Figure S1.** Secondary structure prediction analysis of non-coding regions in the mitochondrial genome of *Hoplolaimus columbus* by FORNA.

## Data Availability

Data supporting the conclusions of this article are included within the article and its additional files. The mitochondrial genome sequence is available in the GenBank database under the accession number MH657221.

## Abbreviations

ML: maximum likelihood phylogenetic analysis
NCR: non-coding region
PCGs: protein coding genes
*rrnS*: *12S* ribosomal RNA
*rrnL*: *16S* ribosomal RNA
*SSU* rDNA: small subunit ribosomal DNA
tRNA: transfer RNA
