# Supplementary material for: The complete mitochondrial genome of the Columbia lance nematode, Hoplolaimus columbus, a major agricultural pathogen in North America
Source: Parasit Vectors. 2020 Jun 22;13:321. doi: 10.1186/s13071-020-04187-y (PMC7310197; doi:10.1186/s13071-020-04187-y)
Supplement: Supplementary file 1 — Additional file 1: Table S1. Model selection for phylogenetic analysis by smart model selection (SMS). Table S2. Microsatellite repeats in intergenic spaces. Table S3. Microsatellite repeats in non-coding regions. Table S4. Tandem repeats in non-coding regions. [file 13071_2020_4187_MOESM1_ESM.docx]

**Additional file 1: Table S1.** Model selection for phylogenetic analysis by Smart Model Selection (SMS)

| **Gene** | **SMS best model with AIC** | **SMS best model with BIC** |
| --- | --- | --- |
| *atp6* | GTR+G+I | GTR+G+I |
| *cox1* | GTR+G+I | GTR+G+I |
| *cox2* | GTR+G+I | GTR+G+I |
| *cox3* | GTR+G+I | GTR+G+I |
| *cytb* | GTR+G+I | GTR+G+I |
| *nad1* | GTR+G+I | GTR+G+I |
| *nad2* | GTR+G+I | GTR+G+I |
| *nad3* | GTR+G+I | GTR+G+I |
| *nad4* | GTR+G+I | GTR+G+I |
| *nad4L* | GTR+G | GTR+G |
| *nad5* | GTR+G+I | GTR+G+I |
| *nad6* | GTR+G+I | GTR+G+I |

**Additional file 1: Table S2.** Microsatellite repeats in intergenic spaces

| Position | Cycle | Rep | Sequence |
| --- | --- | --- | --- |
| >nad3-cox1 | | |  |
| 78 | 2 | 3 | TTTTTT |
| >cox3-trnF | |  |  |
| 14 | 2 | 3 | AAAAAA |
| 187 | 2 | 4 | TTTTTTTT |
| 200 | 2 | 3 | TTTTTT |
| 227 | 2 | 3 | TTTTTT |
| 236 | 2 | 3 | TTTTTT |
| >trnF-nad4 | | |  |
| 213 | 2 | 3 | TTTTTT |
| >trnK-rrnS | |  |  |
| 236 | 2 | 3 | TTTTTT |
| >trnV-trnL1 | | |  |
| 167 | 2 | 3 | TTTTTT |

**Additional file 1: Table S3.** Microsatellite repeats in noncoding regions

| **NCR1** | |  | |  | |  | |  | |  | |  | |  | |  | |  | |  | |  |
| --- | --- | --- | --- | --- | --- | --- | --- | --- | --- | --- | --- | --- | --- | --- | --- | --- | --- | --- | --- | --- | --- | --- |
| Position | | Cycle | | Rep. | | Sequence | | Position | | Cycle | | Rep. | | Sequence | | Position | | Cycle | | Rep. | | Sequence |
| 58 | | 2 | | 4 | | TTTTTTTT | | 2787 | | 2 | | 4 | | TTTTTTTT | | 5254 | | 2 | | 4 | | CTCTCTCT |
| 66 | | 3 | | 3 | | AAAAAAAAA | | 2810 | | 2 | | 3 | | TTTTTT | | 5321 | | 2 | | 3 | | TTTTTT |
| 406 | | 2 | | 3 | | TTTTTT | | 2849 | | 2 | | 3 | | TTTTTT | | 5328 | | 2 | | 3 | | TTTTTT |
| 464 | | 2 | | 3 | | GTGTGT | | 2868 | | 2 | | 3 | | TTTTTT | | 5348 | | 2 | | 3 | | TTTTTT |
| 794 | | 2 | | 4 | | TTTTTTTT | | 2880 | | 2 | | 3 | | TTTTTT | | 5439 | | 2 | | 3 | | TTTTTT |
| 861 | | 2 | | 4 | | TTTTTTTT | | 2888 | | 2 | | 3 | | TTTTTT | | 5464 | | 2 | | 3 | | AGAGAG |
| 971 | | 2 | | 5 | | TTTTTTTTTT | | 2903 | | 2 | | 5 | | AAAAAAAAAA | | 5552 | | 2 | | 3 | | CTCTCT |
| 989 | | 2 | | 4 | | TTTTTTTT | | 2933 | | 2 | | 3 | | GAGAGA | | 5682 | | 2 | | 3 | | CTCTCT |
| 1287 | | 3 | | 3 | | AGGAGGAGG | | 3039 | | 2 | | 3 | | TTTTTT | | 5808 | | 2 | | 3 | | TTTTTT |
| 1377 | | 2 | | 3 | | GAGAGA | | 3058 | | 2 | | 3 | | TTTTTT | | 5832 | | 2 | | 3 | | AAAAAA |
| 1468 | | 2 | | 3 | | AAAAAA | | 3108 | | 2 | | 5 | | TTTTTTTTTT | | 5901 | | 2 | | 3 | | TTTTTT |
| 1583 | | 2 | | 3 | | TTTTTT | | 3179 | | 2 | | 5 | | TTTTTTTTTT | | 5928 | | 2 | | 3 | | TTTTTT |
| 1593 | | 2 | | 3 | | AAAAAA | | 3216 | | 2 | | 3 | | TTTTTT | | 5955 | | 2 | | 5 | | TTTTTTTTTT |
| 1602 | | 2 | | 3 | | TTTTTT | | 3262 | | 2 | | 4 | | TTTTTTTT | | 6097 | | 3 | | 3 | | GAGGAGGAG |
| 1610 | | 2 | | 3 | | TTTTTT | | 3313 | | 2 | | 4 | | TTTTTTTT | | 6210 | | 2 | | 3 | | GAGAGA |
| 1733 | | 2 | | 3 | | TTTTTT | | 3340 | | 3 | | 3 | | ATTATTATT | | 6281 | | 2 | | 3 | | AAAAAA |
| 1835 | | 2 | | 3 | | AAAAAA | | 3395 | | 2 | | 4 | | TTTTTTTT | | 6467 | | 2 | | 3 | | AAAAAA |
| 1888 | | 2 | | 5 | | TTTTTTTTTT | | 3505 | | 2 | | 3 | | TTTTTT | | 6478 | | 2 | | 3 | | CTCTCT |
| 1929 | | 2 | | 4 | | TTTTTTTT | | 3541 | | 2 | | 4 | | TTTTTTTT | | 6490 | | 2 | | 3 | | AGAGAG |
| 1994 | | 2 | | 3 | | TTTTTT | | 3559 | | 2 | | 5 | | TTTTTTTTTT | | 6498 | | 2 | | 3 | | TTTTTT |
| 2011 | | 2 | | 3 | | TTTTTT | | 3577 | | 4 | | 3 | | TATTTATTTATT | | 6661 | | 2 | | 4 | | TTTTTTTT |
| 2068 | | 2 | | 3 | | TTTTTT | | 3606 | | 2 | | 4 | | TTTTTTTT | | 6836 | | 2 | | 4 | | AAAAAAAA |
| 2126 | | 2 | | 4 | | TTTTTTTT | | 3838 | | 3 | | 3 | | TTATTATTA | | 6864 | | 2 | | 4 | | TTTTTTTT |
| 2165 | | 2 | | 4 | | TTTTTTTT | | 3864 | | 2 | | 3 | | TTTTTT | | 6881 | | 2 | | 4 | | AAAAAAAA |
| 2229 | | 2 | | 3 | | TTTTTT | | 4058 | | 2 | | 3 | | TTTTTT | | 6981 | | 2 | | 3 | | AAAAAA |
| 2245 | | 2 | | 3 | | TTTTTT | | 4376 | | 2 | | 3 | | AAAAAA | | 7048 | | 2 | | 3 | | GAGAGA |
| 2303 | | 2 | | 5 | | TTTTTTTTTT | | 4442 | | 2 | | 3 | | CTCTCT | | 7065 | | 2 | | 3 | | TTTTTT |
| 2404 | | 2 | | 3 | | TTTTTT | | 4520 | | 2 | | 3 | | GTGTGT | | 7109 | | 2 | | 3 | | TTTTTT |
| 2449 | | 2 | | 3 | | TTTTTT | | 4627 | | 2 | | 4 | | TTTTTTTT | | 7126 | | 2 | | 3 | | TTTTTT |
| 2495 | | 2 | | 3 | | TTTTTT | | 4642 | | 2 | | 4 | | TTTTTTTT | | 7145 | | 3 | | 3 | | GAGGAGGAG |
| 2540 | | 2 | | 3 | | TTTTTT | | 4690 | | 2 | | 3 | | AGAGAG | | 7320 | | 2 | | 3 | | AAAAAA |
| 2560 | | 2 | | 3 | | TTTTTT | | 4797 | | 2 | | 3 | | TTTTTT | | 7377 | | 2 | | 3 | | AAAAAA |
| 2649 | | 2 | | 3 | | TTTTTT | | 5030 | | 3 | | 3 | | GGAGGAGGA | | 7585 | | 2 | | 3 | | TATATA |
| 2694 | | 2 | | 3 | | AAAAAA | | 5128 | | 2 | | 3 | | GGGGGG | | 7640 | | 2 | | 3 | | GAGAGA |
| 2748 | | 2 | | 4 | | TTTTTTTT | | 5242 | | 2 | | 3 | | TTTTTT | |  | |  | |  | |  |
| **NCR2** | |  | |  | |  | |  | |  | |  | |  | |  | |  | |  | |  |
| Position | Cycle | | Rep | | Sequence | | Position | | Cycle | | Rep | | Sequence | | Position | | Cycle | | Rep | | Sequence | |
| 248 | 2 | | 3 | | TTTTTT | | 1316 | | 2 | | 3 | | TTTTTT | | 2323 | | 2 | | 4 | | TTTTTTTT | |
| 272 | 2 | | 4 | | AAAAAAAA | | 1330 | | 2 | | 3 | | TTTTTT | | 2393 | | 3 | | 3 | | TTATTATTA | |
| 299 | 2 | | 3 | | TTTTTT | | 1378 | | 2 | | 4 | | TTTTTTTT | | 2414 | | 3 | | 4 | | TATTATTATTAT | |
| 308 | 2 | | 3 | | AAAAAA | | 1401 | | 2 | | 5 | | TTTTTTTTTT | | 2428 | | 2 | | 3 | | AAAAAA | |
| 386 | 2 | | 5 | | TTTTTTTTTT | | 1471 | | 2 | | 4 | | TTTTTTTT | | 2449 | | 2 | | 4 | | TTTTTTTT | |
| 397 | 2 | | 3 | | TTTTTT | | 1481 | | 2 | | 4 | | TTTTTTTT | | 2458 | | 2 | | 3 | | TTTTTT | |
| 469 | 2 | | 4 | | TTTTTTTT | | 1509 | | 2 | | 3 | | TTTTTT | | 2479 | | 2 | | 3 | | TTTTTT | |
| 522 | 2 | | 3 | | TTTTTT | | 1553 | | 2 | | 3 | | TTTTTT | | 2494 | | 2 | | 5 | | TTTTTTTTTT | |
| 568 | 2 | | 5 | | TTTTTTTTTT | | 1722 | | 2 | | 5 | | TTTTTTTTTT | | 2506 | | 2 | | 3 | | TTTTTT | |
| 588 | 2 | | 4 | | TTTTTTTT | | 1745 | | 2 | | 3 | | TTTTTT | | 2640 | | 2 | | 4 | | TTTTTTTT | |
| 667 | 2 | | 3 | | TTTTTT | | 1759 | | 2 | | 3 | | TTTTTT | | 2649 | | 2 | | 3 | | ATATAT | |
| 706 | 2 | | 3 | | TTTTTT | | 1768 | | 2 | | 3 | | TTTTTT | | 2690 | | 2 | | 3 | | GGGGGG | |
| 822 | 2 | | 4 | | TTTTTTTT | | 1783 | | 2 | | 6 | | TTTTTTTTTTTT | | 2710 | | 2 | | 4 | | TTTTTTTT | |
| 844 | 2 | | 3 | | TTTTTT | | 1838 | | 2 | | 3 | | TTTTTT | | 2753 | | 2 | | 3 | | TTTTTT | |
| 986 | 2 | | 3 | | TTTTTT | | 1846 | | 2 | | 4 | | TTTTTTTT | | 2871 | | 2 | | 5 | | TTTTTTTTTT | |
| 997 | 2 | | 4 | | TTTTTTTT | | 1957 | | 2 | | 4 | | TTTTTTTT | | 2884 | | 2 | | 4 | | TTTTTTTT | |
| 1006 | 2 | | 4 | | TTTTTTTT | | 1997 | | 2 | | 4 | | TTTTTTTT | | 2940 | | 2 | | 5 | | TTTTTTTTTT | |
| 1060 | 2 | | 3 | | TTTTTT | | 2048 | | 4 | | 3 | | ATTTATTTATTT | | 2952 | | 2 | | 3 | | TTTTTT | |
| 1075 | 3 | | 3 | | TTATTATTA | | 2066 | | 2 | | 4 | | TTTTTTTT | | 2964 | | 2 | | 3 | | TTTTTT | |
| 1088 | 2 | | 3 | | AAAAAA | | 2086 | | 2 | | 3 | | TTTTTT | | 2981 | | 2 | | 3 | | TTTTTT | |
| 1121 | 2 | | 3 | | TTTTTT | | 2105 | | 2 | | 3 | | TTTTTT | | 2988 | | 2 | | 3 | | TTTTTT | |
| 1169 | 2 | | 3 | | TTTTTT | | 2240 | | 2 | | 3 | | CTCTCT | | 3000 | | 2 | | 3 | | TTTTTT | |
| 1235 | 2 | | 4 | | TTTTTTTT | | 2267 | | 2 | | 3 | | TATATA | | 3085 | | 2 | | 3 | | TTTTTT | |
| 1283 | 2 | | 4 | | TTTTTTTT | | 2287 | | 2 | | 3 | | TTTTTT | | 3092 | | 2 | | 4 | | TTTTTTTT | |

**Additional file 1: Table S4.** Tandem repeats in noncoding regions

| NCR1  Indices | Period  Size | Copy  Number | Consensus  Size | Percent  Matches | Percent  Indels | Score | A | C | G | T | Entropy  (0-2) |
| --- | --- | --- | --- | --- | --- | --- | --- | --- | --- | --- | --- |
| 1736--1835 | 52 | 1.9 | 53 | 95 | 2 | 184 | 34 | 2 | 14 | 50 | 1.54 |
| [1789--2308](http://tandem.bu.edu/trf/output/11p3jYh7XZ6uk.s6.2.7.7.80.10.50.500.1.txt.html#1789--2308,238,2.2,233,2) | 238 | 2.2 | 233 | 93 | 3 | 873 | 29 | 4 | 13 | 53 | 1.58 |
| 1789--2309 | 235 | 2.2 | 237 | 93 | 4 | 885 | 28 | 4 | 13 | 53 | 1.58 |
| 2324--2555 | 45 | 5.1 | 45 | 92 | 2 | 376 | 37 | 2 | 12 | 46 | 1.56 |
| 2324--2561 | 91 | 2.6 | 91 | 93 | 2 | 399 | 38 | 2 | 13 | 46 | 1.56 |
| 2497--2609 | 34 | 3.4 | 34 | 68 | 16 | 101 | 34 | 1 | 15 | 48 | 1.55 |
| 2495--2618 | 65 | 1.9 | 63 | 93 | 3 | 212 | 34 | 1 | 15 | 48 | 1.55 |
| 2451--2663 | 110 | 2 | 108 | 92 | 3 | 356 | 36 | 1 | 13 | 47 | 1.54 |
| 2560--2686 | 44 | 2.9 | 44 | 92 | 5 | 220 | 37 | 2 | 13 | 46 | 1.56 |
| 2404--2686 | 154 | 1.8 | 155 | 93 | 3 | 498 | 37 | 2 | 13 | 46 | 1.55 |
| 3982--4121 | 49 | 2.9 | 47 | 77 | 11 | 140 | 20 | 6 | 10 | 62 | 1.49 |
| 5548--5713 | 43 | 3.8 | 43 | 93 | 2 | 287 | 34 | 18 | 21 | 25 | 1.96 |
| 5548--5713 | 87 | 1.9 | 87 | 93 | 2 | 289 | 34 | 18 | 21 | 25 | 1.96 |
|  |  |  |  |  |  |  |  |  |  |  |  |
|  |  |  |  |  |  |  |  |  |  |  |  |
| NCR2  Indices | Period  Size | Copy  Number | Consensus  Size | Percent  Matches | Percent  Indels | Score | A | C | G | T | Entropy  (0-2) |
| 571--607 | 18 | 2.2 | 17 | 86 | 13 | 51 | 27 | 0 | 0 | 72 | 0.84 |
| 981--1040 | 21 | 2.8 | 20 | 80 | 7 | 57 | 16 | 1 | 6 | 75 | 1.1 |
| 2069--2112 | 18 | 2.4 | 19 | 88 | 3 | 63 | 11 | 4 | 6 | 77 | 1.11 |
| 2864--2914 | 23 | 2.2 | 23 | 78 | 0 | 57 | 15 | 0 | 9 | 74 | 1.06 |
| 2977--3028 | 19 | 2.7 | 20 | 79 | 8 | 54 | 19 | 1 | 5 | 73 | 1.14 |
