# Supplementary material for: The complete mitochondrial genome of the Columbia lance nematode, Hoplolaimus columbus, a major agricultural pathogen in North America
Source: Parasit Vectors. 2020 Jun 22;13:321. doi: 10.1186/s13071-020-04187-y (PMC7310197; doi:10.1186/s13071-020-04187-y)

**Additional file 2: Figure S1.** Secondary structure prediction analysis of non-coding regions (NCR) in the mitochondrial genome of *Hoplolaimus columbus* by FORNA

NCR1


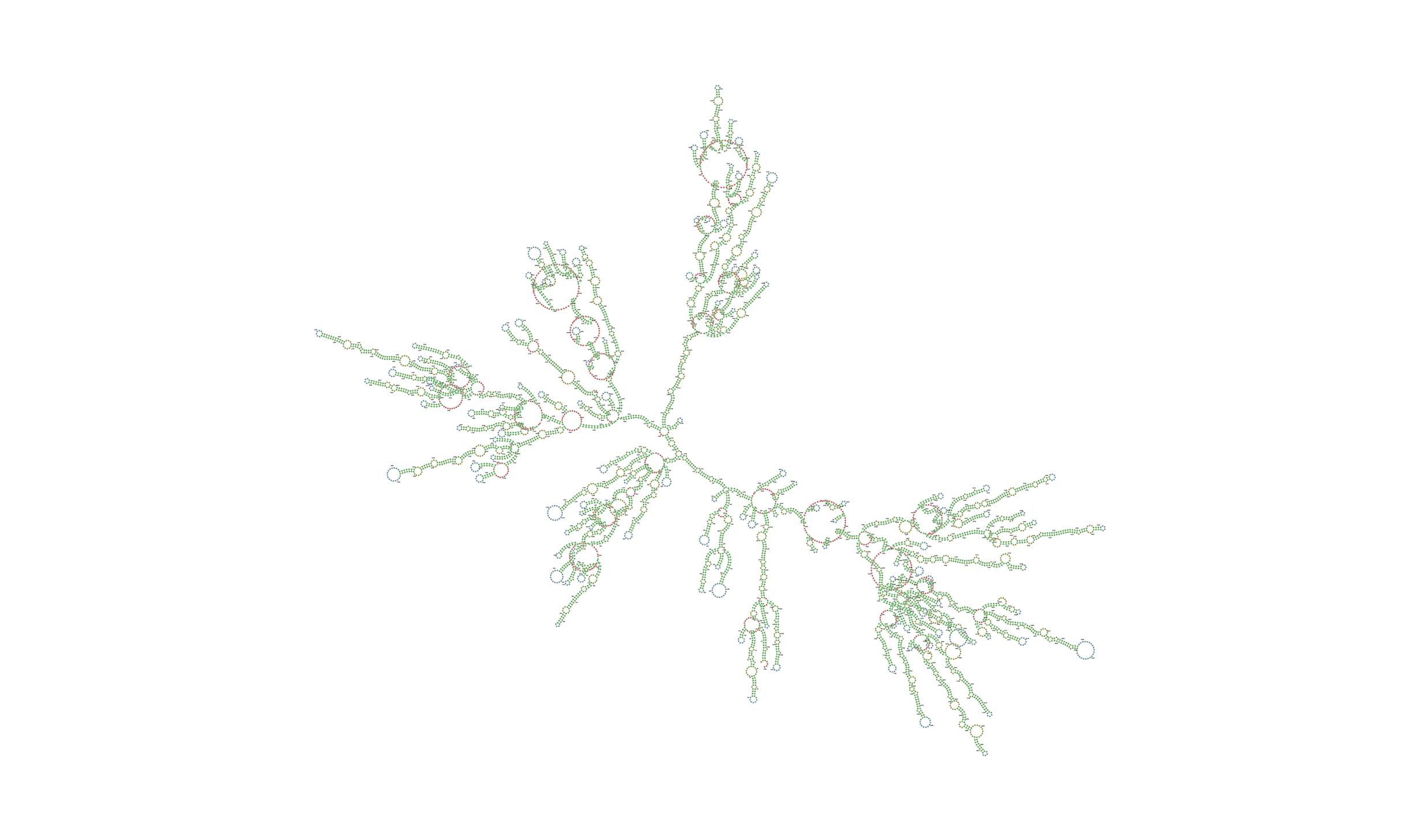


NCR2


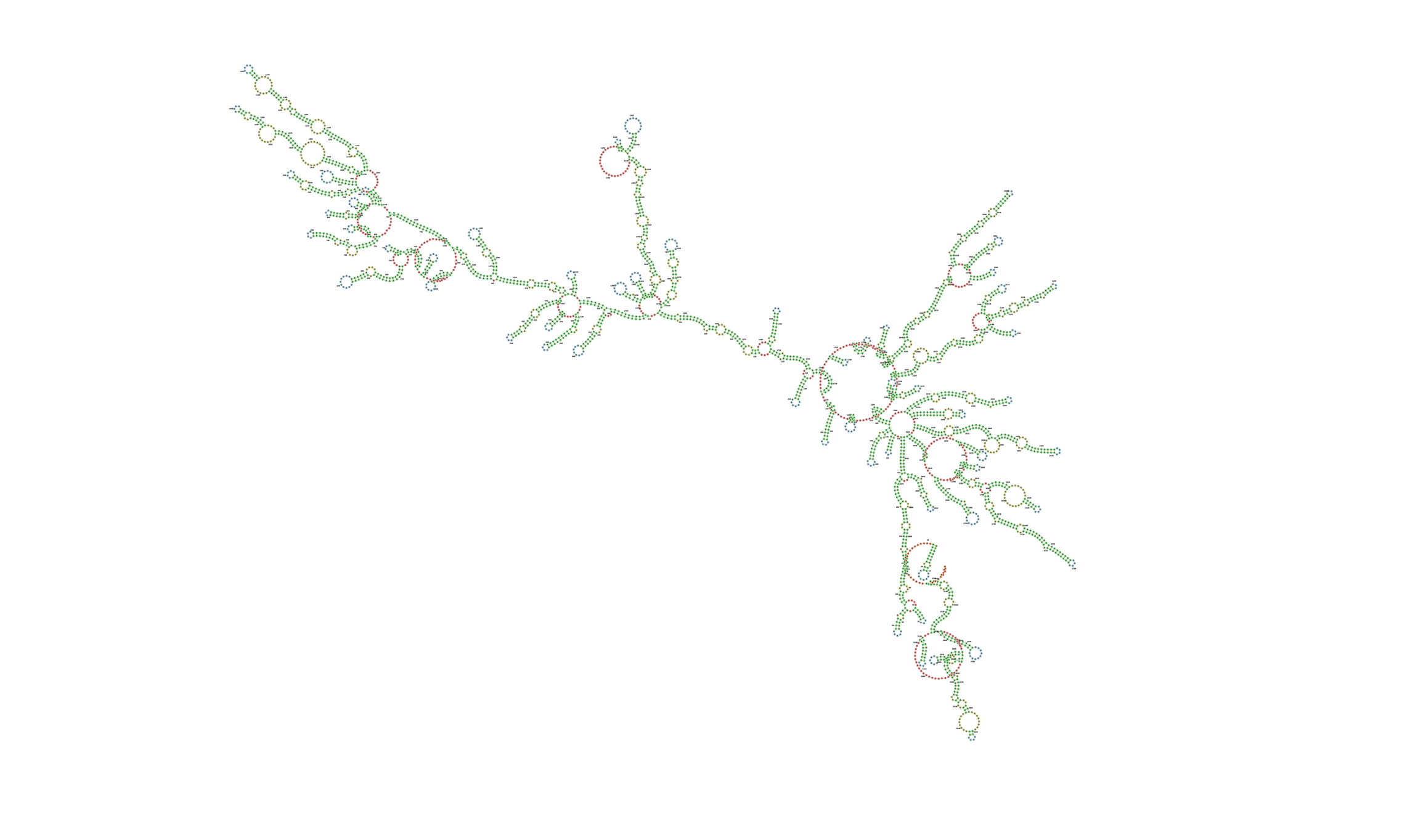

Supplement: Supplementary file 2 — Additional file 2: Figure S1. Secondary structure prediction analysis of non-coding regions in the mitochondrial genome of Hoplolaimus columbus by FORNA. [file 13071_2020_4187_MOESM2_ESM.docx]
